# Equivalence of entropy balancing and the method of moments for matching‐adjusted indirect comparison

**DOI:** 10.1002/jrsm.1416

**Published:** 2020-05-27

**Authors:** David M. Phillippo, Sofia Dias, A. E. Ades, Nicky J. Welton

**Affiliations:** ^1^ Bristol Medical School (Population Health Sciences) University of Bristol Bristol UK; ^2^ Centre for Reviews and Dissemination University of York York UK

**Keywords:** effect modification, indirect comparison, individual patient data, matching‐adjusted indirect comparison, population adjustment

## Abstract

Indirect comparisons are used to obtain estimates of relative effectiveness between two treatments that have not been compared in the same randomized controlled trial, but have instead been compared against a common comparator in separate trials. Standard indirect comparisons use only aggregate data, under the assumption that there are no differences in effect‐modifying variables between the trial populations. Population‐adjusted indirect comparisons aim to relax this assumption by using individual patient data (IPD) from one trial to adjust for differences in effect modifiers between populations. At present, the most commonly used approach is matching‐adjusted indirect comparison (MAIC), where weights are estimated that match the covariate distributions of the reweighted IPD to the aggregate trial. MAIC was originally proposed using the method of moments to estimate the weights, but more recently entropy balancing has been proposed as an alternative. Entropy balancing has an additional “optimality” property ensuring that the weights are as uniform as possible, reducing the standard error of the estimates. In this brief method note, we show that MAIC weights are mathematically identical whether estimated using entropy balancing or the method of moments. Importantly, this means that the standard MAIC (based on the method of moments) also enjoys the “optimality” property. Moreover, the additional flexibility of entropy balancing suggests several interesting avenues for further research, such as combining population adjustment via MAIC with adjustments for treatment switching or nonparametric covariate adjustment.

## 1 INTRODUCTION

Estimates of relative treatment effects are required for health care decision‐making, for example, in health technology assessment or regulatory/reimbursement decisions. A common scenario encountered is where two treatments of interest, say *B* and *C*, have not been compared head‐to‐head in the same randomized controlled trial, but instead are compared against a common comparator *A* in separate *AB* and *AC* trials. In such scenarios, an indirect comparisons^1^ may be used to obtain an estimate of the relative effect of *C* vs *B*, denoted *d*_BC_, by comparing the relative effect estimates reported in the *AB* and *AC* trials as ${\hat{d}}_{\mathrm{BC}}={\hat{d}}_{\mathrm{AC}}-{\hat{d}}_{\mathrm{AB}}$ (on a suitable scale, eg, log odds ratios, log hazard ratios, or mean differences). However, if there are differences in effect‐modifying variables between the two study populations, this indirect comparison will be biased.^2^, ^3^ If individual patient data (IPD) are available from both the *AB* and *AC* study, standard regression or weighting methods may be used to adjust for differences in effect‐modifying variables between the study populations. However, it is common for IPD to only be available from one study and published aggregate data from the other. For example, in health technology assessment a company submits evidence of clinical and cost effectiveness to a reimbursement body such as the National Institute for Health and Care Excellence in England and Wales. The submitting company will typically have IPD from their own trial (say *AB*), but only published aggregate data from their competitor's trial (*AC*).

Methods for population‐adjusted indirect comparison have been proposed that aim to adjust for any differences in observed effect modifiers between populations, using IPD from one study and aggregate data from another.^2^, ^3^ At present, the most commonly used approach^2^, ^4^ is *matching‐adjusted indirect comparison* (MAIC).^5^ MAIC is a weighting approach, where weights *w*_*ik*_ are estimated so that the weighted covariate distribution in the *AB* study matches that of the *AC* study. Using these weights, mean outcome on treatments *k* = *A*, *B* in the *AC* population are estimated by taking a weighted average of the outcomes *y*_*ik*(*AB*)_ of the *N*_*k*(*AB*)_ individuals *i* on treatment *k* in the *AB* population

(1) $${\hat{y}}_{k\left(\mathrm{AC}\right)}=\frac{\sum_{i=1}^{N_{k\left(\mathrm{AB}\right)}}y_{\mathrm{ik}\left(\mathrm{AB}\right)}w_{\mathrm{ik}}}{\sum_{i=1}^{N_{k\left(\mathrm{AB}\right)}}w_{\mathrm{ik}}}.$$

A population‐adjusted indirect comparison is then constructed in the *AC* study population as

(2) $${\hat{d}}_{\mathrm{BC}\left(\mathrm{AC}\right)}={\hat{d}}_{\mathrm{AC}\left(\mathrm{AC}\right)}-{\hat{d}}_{\mathrm{AB}\left(\mathrm{AC}\right)},$$

where ${\hat{d}}_{\mathrm{AB}\left(\mathrm{AC}\right)}=g({\hat{y}}_{B\left(\mathrm{AC}\right)})-g({\hat{y}}_{A\left(\mathrm{AC}\right)})$ for a suitable link function *g*(·), and ${\hat{d}}_{\mathrm{AC}\left(\mathrm{AC}\right)}$ is reported by the *AC* study.

Signorovitch et al^5^ proposed to estimate the weights *w*_*ik*_ using the method of moments to balance the mean covariate values (and any included higher order terms, for example squared covariate values to balance the variance) between the weighted *AB* population and the *AC* population. Belger et al^6^, ^7^ suggest another form of population reweighting based on entropy balancing,^8^ which matches moments of the covariate distributions under the additional constraint that the optimal entropy balancing weights are those which are as close as possible to uniform weights (ie, as close as possible to no weighting at all). This additional constraint means that entropy balancing methods should (at least for homoskedastic outcomes) have equal or reduced SE (and equal or greater effective sample size) compared to MAIC, while achieving the same reduction in bias. However, as we now show, estimation of weights via entropy balancing and the method of moments are in fact entirely equivalent. This leads to an important conclusion regarding the optimality of standard MAIC weights based on the method of moments, and suggests interesting avenues for further research.

## 2 EQUIVALENCE OF THE METHOD OF MOMENTS AND ENTROPY BALANCING

The estimation of weights for MAIC, whether based on the method of moments or on entropy balancing, can be formulated as a minimization problem.^5^, ^8^ Equivalence therefore follows from consideration of the respective objective functions that are to be minimized.

Let ***x***_*ik*_ be a vector of covariate values for an individual *i* on treatment *k* in the *AB* study. Signorovitch et al^5^ showed that, after centering the covariates around the means in the *AC* study (ie, so that ${\bar{x}}_{\mathrm{AC}}=0$), MAIC minimizes the objective function

(3) $$H_{\mathrm{MM}}\left(\alpha \right)=\sum_{k=A,B}\sum_{i=1}^{N_{k\left(\mathrm{AB}\right)}}\exp\left(x_{\mathrm{ik}}^{T}\alpha \right),$$

for a vector of parameters ***α***. With solution $\hat{\alpha }=\arg\;\min\left(H_{\mathrm{MM}}\left(\alpha \right)\right)$, the (normalized) weights *w*_*ik*_ are then given by

(4) $$w_{\mathrm{ik}}=\frac{\exp\left(x_{\mathrm{ik}}^{T}\hat{\alpha }\right)}{\sum_{v=A,B}\sum_{u=1}^{N_{v\left(\mathrm{AB}\right)}}\exp\left(x_{\mathrm{uv}}^{T}\hat{\alpha }\right)}.$$

(We use the normalized weights here to better show the equivalence to entropy balancing; a set of weights can be rescaled arbitrarily without affecting the estimate in Equation (1).^2^, ^5^)

Entropy balancing also seeks weights that match the moments of covariates between studies, but that further minimize the entropy distance from uniform weights, $\sum_{k=A,B}\sum_{i=1}^{N_{k\left(\mathrm{AB}\right)}}w_{\mathrm{ik}}\log\left(N_{\left(\mathrm{AB}\right)}w_{\mathrm{ik}}\right)$. Hainmueller^8^ used Lagrange multipliers to find an unconstrained dual optimization problem, which (again after setting ${\bar{x}}_{\mathrm{AC}}=0$) gives the objective function

(5) $$H_{\mathrm{EB}}\left(\alpha \right)=\log\left(\frac{1}{N_{\left(\mathrm{AB}\right)}}\sum_{k=A,B}\sum_{i=1}^{N_{k\left(\mathrm{AB}\right)}}\exp\left(x_{\mathrm{ik}}^{T}\alpha \right)\right).$$

With solution $\hat{\alpha }=\arg\;\min\left(H_{\mathrm{EB}}\left(\alpha \right)\right)$, the weights are again given by (4).

Comparing the objective functions (3) and (5), we see that

(6) $$H_{\mathrm{EB}}\left(\alpha \right)=\log\left(H_{\mathrm{MM}}\left(\alpha \right)\right)-\log\left(N_{\left(\mathrm{AB}\right)}\right).$$

Therefore, since the logarithm is a monotonic function and log(*N*_(*AB*)_) is constant, the solutions of these two minimization problems are identical; MAIC weights based on the method of moments or entropy balancing are identical up to a normalizing constant.

Example R code is provided in the Appendix S1 that implements both the method of moments and entropy balancing approaches to MAIC, applied to the simulated example given by Phillippo et al.^2^

## 3 DISCUSSION

In this brief method note, we have shown that the MAIC weights are identical whether estimated using entropy balancing or the method of moments. In practice, entropy balancing performs the minimization on the log scale which may perform better computationally, but the estimated weights will be identical for MAIC and entropy balancing, up to optimization error. An important corollary from this result is that standard MAIC (based on the method of moments) also enjoys the additional “optimality” property that the estimated weights are as close as possible to uniform weights (no weighting at all), in an entropy sense. Alternative loss functions could be used in the entropy balancing scheme which may change the performance of the method, and would then no longer be equivalent to standard MAIC based on the method of moments. For example, it remains to be seen whether other loss functions could be used to obtain MAIC weights that are optimal in the sense that they minimize the SE of the resulting population‐adjusted estimates (or equivalently, maximize the effective sample size); this is likely of greater practical interest than pursuing optimality in the entropy sense.

For entropy balancing, Hainmueller^8^ notes that other “base weights” for which to minimise the distance from could be used instead of uniform weights, and this would also depart from equivalence to standard MAIC based on the method of moments. With non‐uniform base weights $w_{\mathrm{ik}}^{\left(0\right)}$, the entropy balancing objective function in (5) becomes

(7a) $$H_{\mathrm{EB}}\left(\alpha \right)=\log\left(\sum_{k=A,B}\sum_{i=1}^{N_{k\left(\mathrm{AB}\right)}}w_{\mathrm{ik}}^{\left(0\right)}\exp\left(x_{\mathrm{ik}}^{T}\alpha \right)\right),$$

and the weights are then given by

(7b) $$w_{\mathrm{ik}}=\frac{w_{\mathrm{ik}}^{\left(0\right)}\exp\left(x_{\mathrm{ik}}^{T}\hat{\alpha }\right)}{\sum_{v=A,B}\sum_{u=1}^{N_{v\left(\mathrm{AB}\right)}}w_{\mathrm{uv}}^{\left(0\right)}\exp\left(x_{\mathrm{uv}}^{T}\hat{\alpha }\right)}.$$

Setting uniform base weights $w_{\mathrm{ik}}^{\left(0\right)}=1/N_{\left(\mathrm{AB}\right)}$ in (7) recovers formula (5) above. Non‐uniform base weights could, for example, be used to perform nonparametric covariate adjustment,^9^ or to adjust for treatment switching,^10^ prior to population adjustment by weighting to match the *AC* population. The idea is that the final weights aim to retain the initial adjustment applied by the base weights, while also applying the necessary population adjustment. This would be a novel development for MAIC, and is an interesting avenue for further research. It remains to be seen how this approach might perform in practice, for example, if the population differences are large and the final weights are far from the base weights. The example R code in the Appendix S1 also includes an implementation of entropy balancing MAIC with non‐uniform base weights.

Different schemes for applying weights have also been proposed. MAIC as described by Signorovitch et al^5^ estimates weights for the entire *AB* population at once to balance covariate distributions with the entire *AC* population. Belger et al^6^, ^7^ compare with other possible approaches, which involve splitting apart trial arms and balancing covariate distributions separately between the control arms (*A*) and between the treatment arms (*B* and *C*) in the IPD and aggregate populations. The properties of such “splitting” approaches in comparison with a more typical population reweighting are largely unknown and require further investigation; however, some initial simulation studies have reported performance benefits over standard MAIC.^11^ While MAIC is at present the most commonly used approach for population adjustment, other methods are available which may have advantages over MAIC.^2^, ^12^, ^13^ Recent simulation work showed that regression‐based approaches such as multilevel network meta‐regression and simulated treatment comparison performed better than MAIC in many scenarios, and that in some cases MAIC could even increase bias compared to a standard indirect comparison.^12^

We have discussed an “anchored” indirect comparison scenario where a common comparator arm is available. However, a sizeable proportion of MAIC analyses published to date instead rely on an “unanchored” indirect comparison, where absolute outcomes on treatments *B* and *C* from single‐arm studies or in a disconnected network are compared directly as ${\hat{d}}_{\mathrm{BC}\left(C\right)}=g({\hat{y}}_{C\left(C\right)})-g({\hat{y}}_{B\left(C\right)})$, where ${\hat{y}}_{B\left(C\right)}$ is estimated using weights and ${\hat{y}}_{C\left(C\right)}$ is reported by the *C* trial.^2^, ^4^ Unanchored comparisons rely on a much stronger assumption than anchored comparisons, namely that all prognostic factors as well as all effect modifiers have been suitably adjusted for.^2^, ^3^ The equivalence of the method of moments and entropy balancing approaches follows in exactly the same manner in an unanchored setting. Unanchored MAICs have previously been used in scenarios with a common comparator but where treatment switching is present.^2^, ^4^ The entropy balancing approach with non‐uniform base weights, described above, provides an attractive option for combining weight‐based adjustments for treatment switching^10^ with an anchored MAIC, while crucially retaining reliance on randomization.

Several simulation studies have compared approaches based on standard MAIC and entropy balancing and found no difference between these approaches.^6^, ^7^, ^11^ The equivalence result given in this paper explains these findings, as we now know that these approaches are identical up to the numerical accuracy of the optimization routines. Available guidance on the use of MAIC (eg, ^2^) should be updated to note the equivalence of entropy balancing and standard MAIC.

In conclusion, the equivalence of MAIC weights estimated using the method of moments and entropy balancing means that standard MAIC (based on the method of moments) inherits the desirable “optimality” property that the weights are as uniform as possible. Moreover, the additional flexibility of entropy balancing suggests several interesting avenues for further research.

## CONFLICT OF INTEREST

D.M.P. reports personal fees from UCB outside of the submitted work.

## Supporting information


**Appendix** S1. Example R code implementing MAIC using the method of moments (3) and using entropy balancing with uniform base weights (5) and non‐uniform base weights (7).

## Data Availability

Data sharing is not applicable to this article as no new data were created or analysed.
